# Supplementary material for: Stem cell factor is selectively secreted by arterial endothelial cells in bone marrow
Source: Nat Commun. 2018 Jun 22;9:2449. doi: 10.1038/s41467-018-04726-3 (PMC6015052; doi:10.1038/s41467-018-04726-3)
Supplement: Supplementary file 2 — Supplementary Information [file 41467_2018_4726_MOESM2_ESM.pdf]

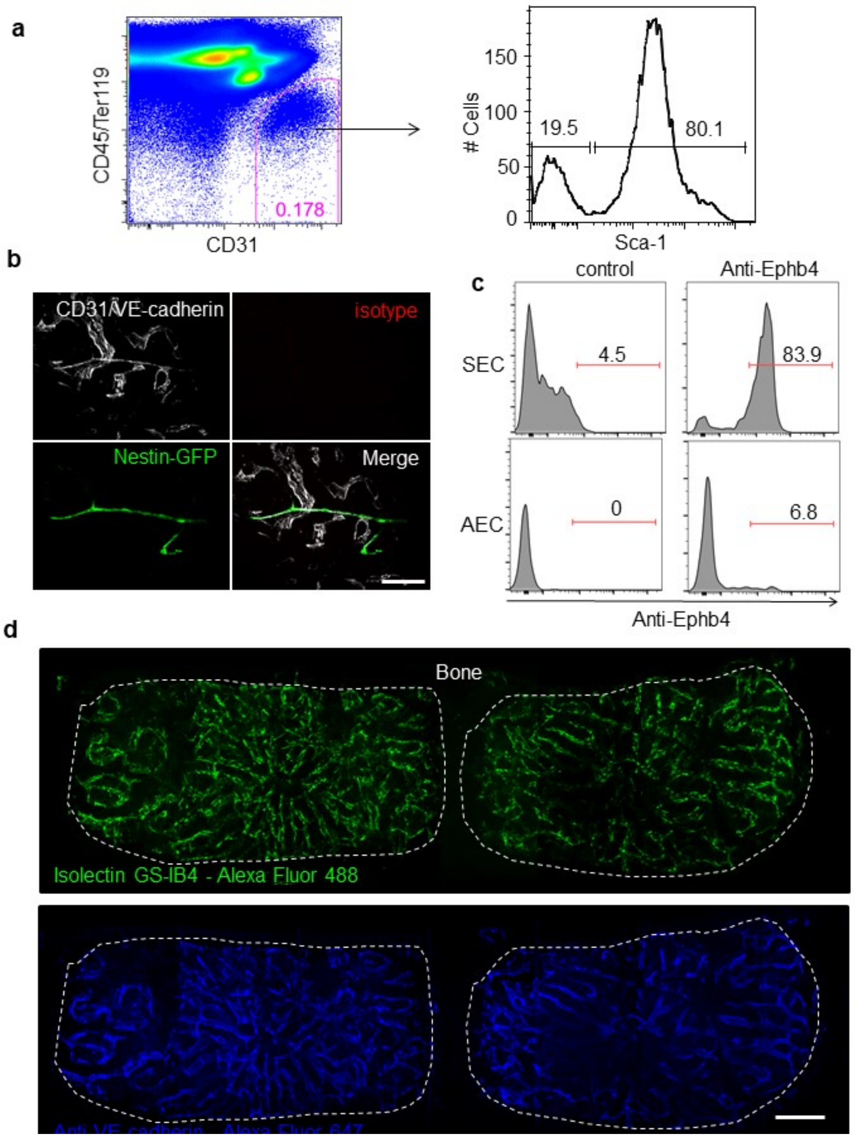

Supplementary Figure 1. FACS analysis of Sca-1 expression on CD45⁻ Ter119⁻ CD31⁺ cells and whole-mount imaging of mouse sternum endothelial cells stained *in vivo*. (a) Representative FACS plot of Sca-1 expression on bone marrow CD31⁺ CD45⁻Ter119⁻ cells. Cells were pre-gated on singlet, live cells. (b) Representative imaging of femur BM from *Nestin*-GFP mice stained with anti-VE-cadherin-Alexa fluor 647 and IgG control antibody by *i.v.* Scale bar, 50 µm. c) Representative FACS plot of Ephb4 expression on bone marrow AECs and SECs. d) Wild type mice were injected *i.v.* with Isolectin GS-IB4-Alexa fluor 488 and anti-VE-cadherin-Alexa fluor 647, and the sternal bones were removed for the whole-mount imaging. Shown are two sternal segments. Scale bar, 200 µm.

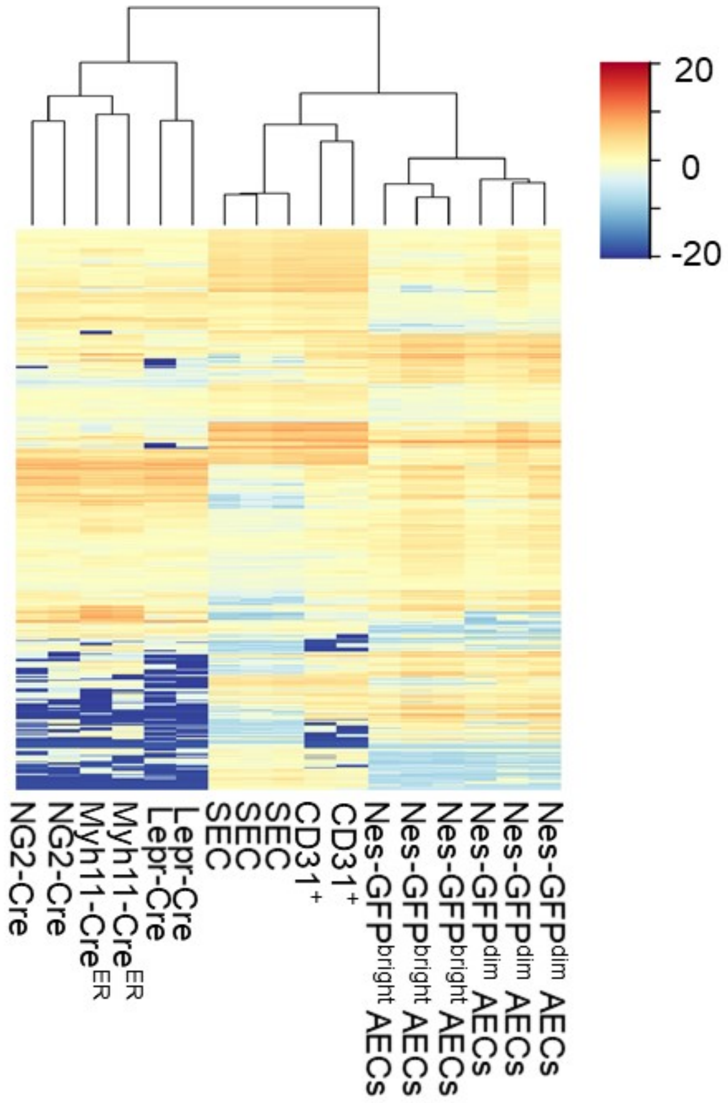

Supplementary Figure 2. Hierarchical clustering analysis of gene expression by different niche cell types. RNAseq data of stromal cells (*NG2*-Cre, *Myh11*-CreER, *Lepr*-Cre) and CD31$^+$ endothelial cells (stained by *i.v.* injection) were from our previous publication[10].

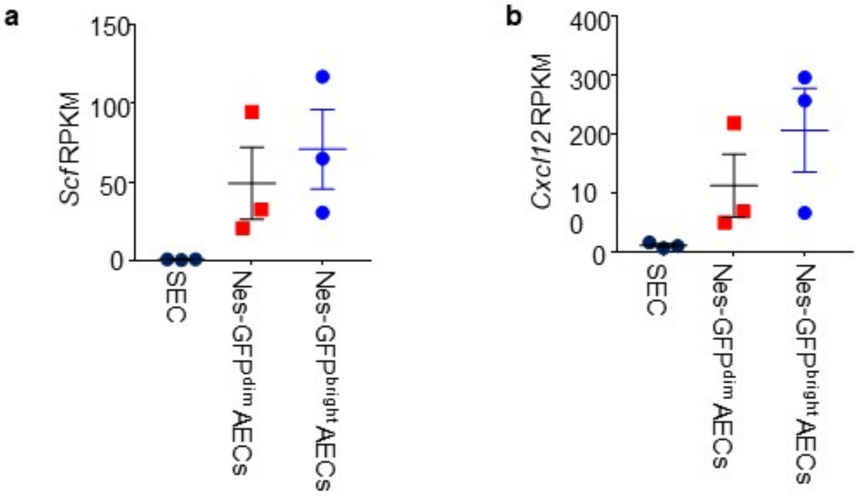

Supplementary Figure 3. AECs express higher *Scf* and *Cxcl12*. RPKM values of *Scf* (a) and *Cxcl12* (b) in SECs, *Nestin*-GFP^{dim} AECs and *Nestin*-GFP^{bright} AECs. n = 3 for each group.

a

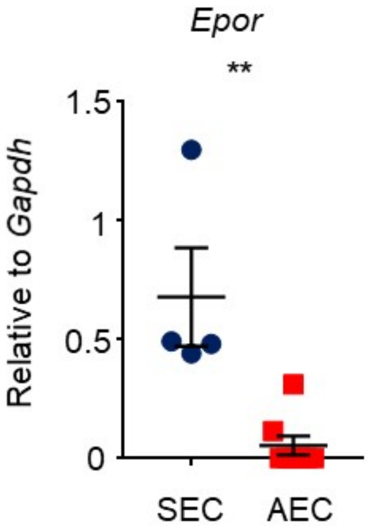

b

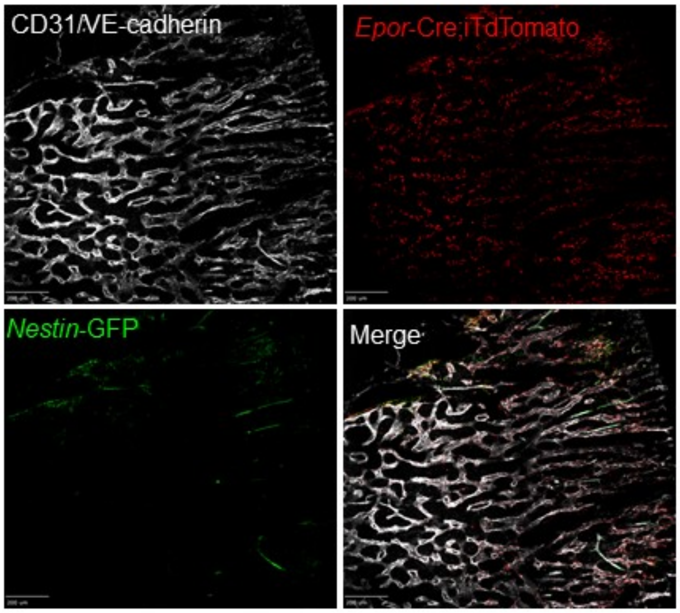

Supplementary Figure 4. Epor expression in bone marrow endothelial cells. (a) qPCR analysis of *Epor* mRNA in sorted AECs and SECs. Data are represented as mean ± SEM. Data were analysed with two-tailed *t*-test. **p < 0.01. n = 4 for each group. (b) Image of tibia metaphysis from *Epor*-Cre;iTdTomato;*Nestin*-GFP mice.

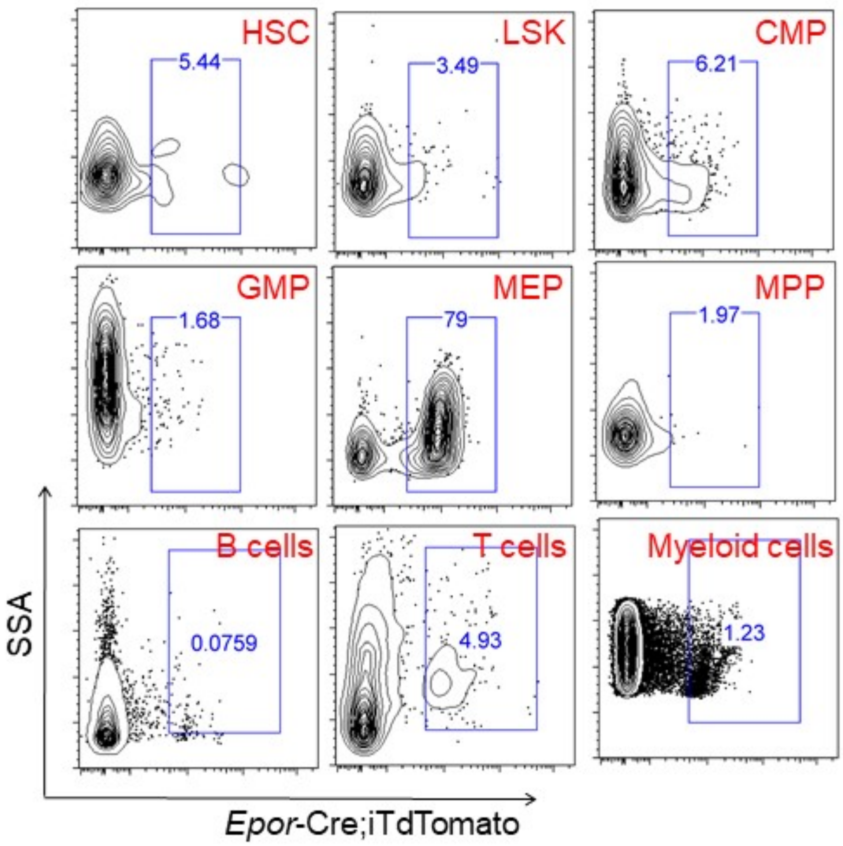

Supplementary Figure 5. *Epor*-Cre labels MEP in hematopoietic stem/progenitor cells compartment. LSK: lineage^{-} Sca-1^{+} c-kit^{+}; HSC: CD150^{+} CD48^{-} LSK; MPP: multipotent progenitor cells, CD150^{-} CD48^{-} LSK; MEP: megakaryocyte-erythroid progenitors, Lin^{-} Sca-1^{-} c-Kit^{+} CD34^{-} FcγR^{-}; CMP: common myeloid progenitors, Lin^{-} Sca-1^{-} c-Kit^{+} CD34^{+} FcγR^{-}; GMP: granulocyte-macrophage progenitor, Lin^{-} Sca-1^{-} c-Kit^{+} CD34^{+} FcγR^{+}; B cells: B220^{+} CD3^{-} cells; T cells: B220^{-} CD3^{+} cells; myeloid cells: CD11b^{+} Gr1^{+} cells.

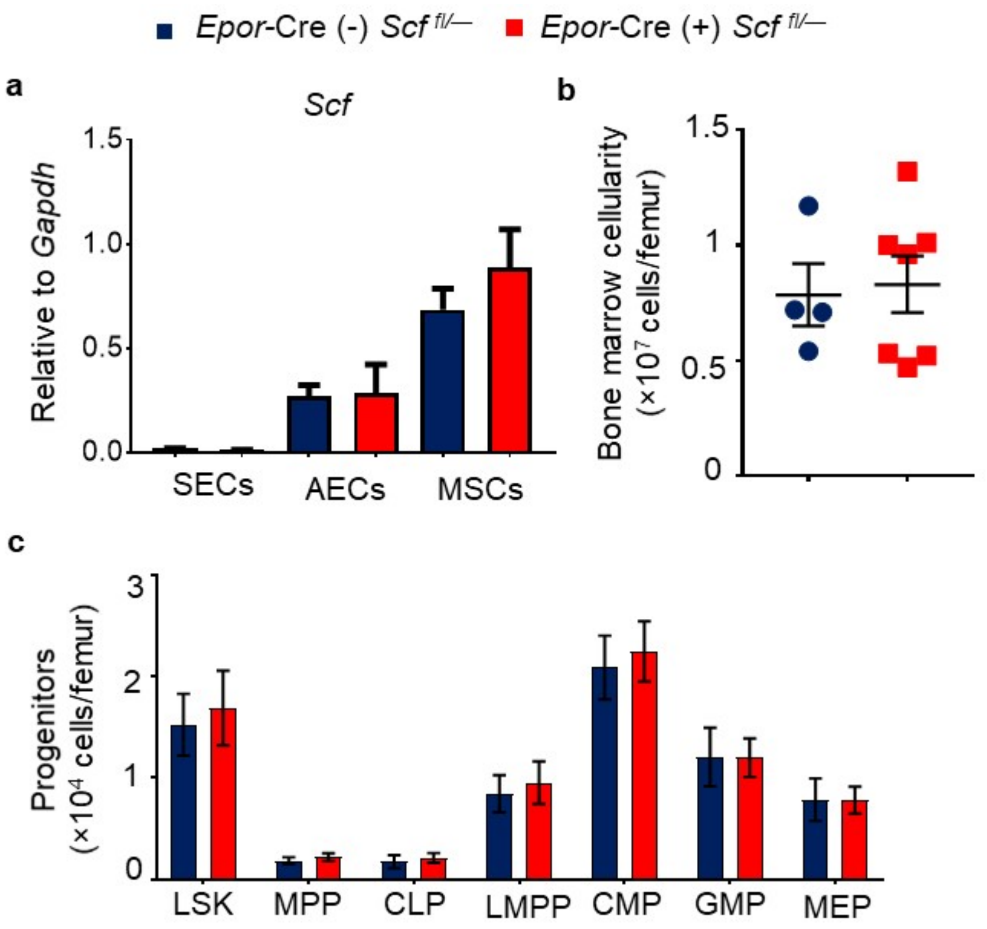

Supplementary Figure 6. Characterization of *Epor*-Cre; *Scf* $^{fl/—}$ mice. *(a) Scf* mRNA level in SECs, AECs, and MSCs from *Epor*-Cre (-) *Scf* $^{fl/—}$ and *Epor*-Cre (+) *Scf* $^{fl/—}$ mice. n = 5 mice for *Epor*-Cre (-) *Scf* $^{fl/—}$; n = 4 mice for *Epor*-Cre (+) *Scf* $^{fl/—}$. (b) Bone marrow cellularity. Data are represented as mean ± SEM. n = 4 mice for *Epor*-Cre (-) *Scf* $^{fl/—}$; n = 7 mice for *Epor*-Cre (+) *Scf* $^{fl/—}$.(c) Absolute numbers of hematopoietic progenitors in the bone marrow. LSK: lineage$^{—}$ Sca-1^{+} c-kit^{+}; MPP: multipotent progenitor cells, CD150$^{—}$ CD48$^{—}$ LSK; MEP: megakaryocytic/erythroid progenitors, Lin$^{—}$ Sca-1$^{—}$ c-Kit^{+} CD34$^{—}$ FcγR$^{—}$; CMP: common myeloid progenitors, Lin$^{—}$ Sca-1$^{—}$ c-Kit^{+} CD34^{+} FcγR$^{—}$; GMP: granulocyte-macrophage progenitor, Lin$^{—}$ Sca-1$^{—}$ c-Kit^{+} CD34^{+} FcγR^{+}; LMPP: lymphoid-primed multipotent progenitor, FLT3^{+} LSK ; CLP: common lymphoid progenitor, Lin$^{—}$ Sca-1^{low} c-Kit^{low} FLT3^{+} IL7Rα^{+}. Data are represented as mean ± SEM. n = 4 mice for *Epor*-Cre (-) *Scf* $^{fl/—}$; n = 7 mice for *Epor*-Cre (+) *Scf* $^{fl/—}$.

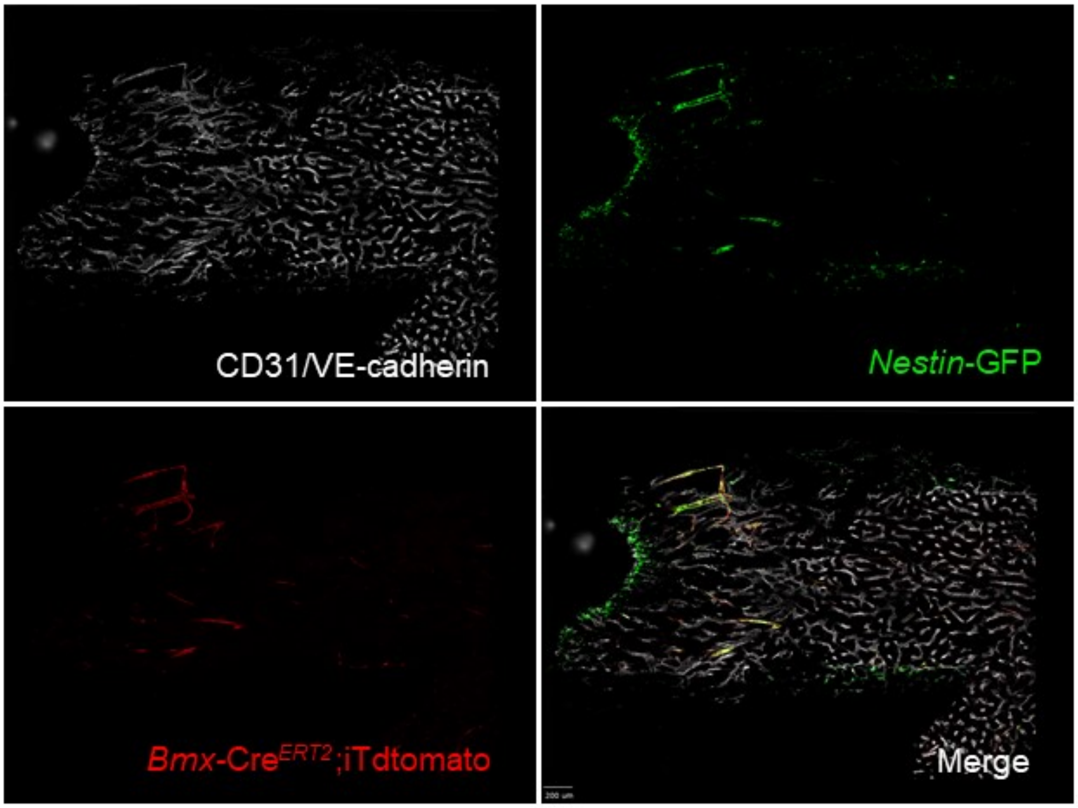

Supplementary Figure 7. Representative image of femur metaphysis from *Bmx*-Cre^{ERT2};iTdtomato;*Nestin*-GFP mice. *Bmx*-Cre^{ERT2};iTdtomato;*Nestin*-GFP mice were stained with anti-VE-cadherin *i.v.* and femur was removed for imaging analysis. All the panels show the same field for different channels. Scale bar, 200 μm.

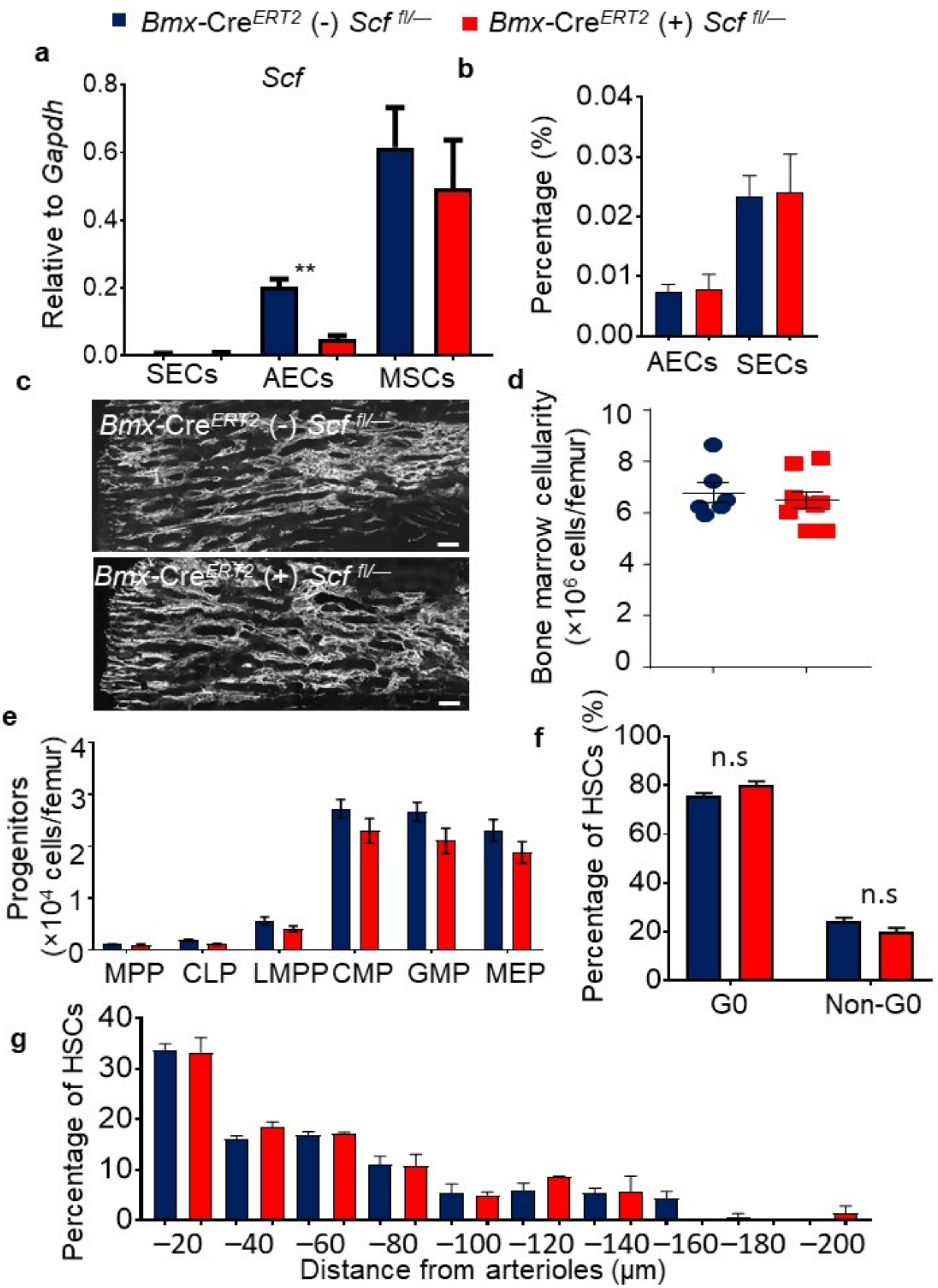

Bmx-Cre^{ERT2} (-) Scf $^{fl/—}$
Bmx-Cre^{ERT2} (+) Scf $^{fl/—}$
a
Scf
Relative to Gapdh
0.0
0.2
0.4
0.6
0.8
**
SECs
AECs
MSCs
b
Percentage (%)
0.00
0.01
0.02
0.03
0.04
AECs
SECs
c
Bmx-Cre^{ERT2} (-) Scf $^{fl/—}$
Bmx-Cre^{ERT2} (+) Scf $^{fl/—}$
d
Bone marrow cellularity
(×10^6 cells/femur)
0
2
4
6
8
10
e
Progenitors
(×10^4 cells/femur)
0
1
2
3
4
MPP
CLP
LMPP
CMP
GMP
MEP
f
Percentage of HSCs (%)
0
20
40
60
80
100
n.s
n.s
G0
Non-G0
g
Percentage of HSCs
0
10
20
30
40
−20
−40
−60
−80
−100
−120
−140
−160
−180
−200
Distance from arterioles (μm)

Supplementary Figure 8. Characterization of *Bmx*- Cre^{ERT2}(+) *Scf* $^{fl/—}$ mice. (a) *Scf* mRNA level in SECs, AECs, and MSCs from *Bmx*-Cre^{ERT2} (-) *Scf* $^{fl/—}$ and *Bmx*-Cre^{ERT2} (+) *Scf* $^{fl/—}$ mice. n = 4 mice for *Bmx*-Cre^{ERT2} (-) *Scf* $^{fl/—}$; n = 3 mice for *Bmx*-Cre^{ERT2} (+) *Scf* $^{fl/—}$. (b) Percentage of AECs and SECs among bone marrow mononuclear cells. n = 4 mice for *Bmx*-Cre^{ERT2} (-) *Scf* $^{fl/—}$; n = 3 mice for *Bmx*-Cre^{ERT2} (+) *Scf* $^{fl/—}$. (c) Immunofluorescence microscopy imaging of tibia from *Bmx*-*C*re^{ERT2} (-) *Scf* $^{fl/—}$ and for *Bmx*-Cre^{ERT2} (+) *Scf* $^{fl/—}$. Scale bar, 100 um. (d) Bone marrow cellularity. Data are represented as mean ± SEM. n = 6 mice for *Bmx*-Cre^{ERT2} (-) *Scf* $^{fl/—}$; n = 9 mice for *Bmx*-Cre^{ERT2} (+) *Scf* $^{fl/—}$. (e) Absolute numbers of hematopoietic progenitors in the bone marrow. Data are represented as mean ± SEM. n = 6 mice for *Bmx*-Cre^{ERT2} (-) *Scf* $^{fl/—}$; n = 9 mice for *Bmx*-Cre^{ERT2} (+) *Scf* $^{fl/—}$. (f) Quantification of cell cycle of HSCs with Ki-67 and Hoechst 33342 staining. n = 4 mice for *Bmx*-Cre^{ERT2} (-) *Scf* $^{fl/—}$; n = 3 mice for *Bmx*-Cre^{ERT2} (+) *Scf* $^{fl/—}$. (g) HSC localization relative to arterioles. HSCs number = 125 for *Bmx*-Cre^{ERT2} (-) *Scf* $^{fl/—}$ mice and 59 for *Bmx*-Cre^{ERT2} (+) *Scf* $^{fl/—}$ mice.
